# Supplementary material for: Impaired lipid biosynthesis hinders anti-tumor efficacy of intratumoral iNKT cells
Source: Nat Commun. 2020 Jan 23;11:438. doi: 10.1038/s41467-020-14332-x (PMC6978340; doi:10.1038/s41467-020-14332-x)
Supplement: Supplementary file 1 — Supplementary Information [file 41467_2020_14332_MOESM1_ESM.pdf]

Supplementary Information for

Impaired lipid biosynthesis hinders anti-tumor efficacy of intratumoral iNKT cells

Fu et al.

Supplementary Methods

qPCR primers for mouse genes are listed as follows: *Cpt1a* forward, AGA TCA ATC GGA CCC TAG ACA C; reverse, CAG CGA GTA GCG CAT AGT CA; *Srebf1* forward, GCA GCC ACC ATC TAG CCT G; reverse, CAG CAG TGA GTC TGC CTT GAT; *Srebf2* forward, GCA GCA ACG GGA CCA TTC T; reverse, CCC CAT GAC TAA GTC CTT CAA CT; *Acaca* forward, ATG GGC GGA ATG GTC TCT TTC; reverse, TGG GGA CCT TGT CTT CAT CAT; *Fasn* forward, GGA GGT GGT GAT AGC CGG TAT; reverse, TGG GTA ATC CAT AGA GCC CAG; *Hmgcr* forward, AGC TTG CCC GAA TTG TAT GTG; reverse, TCT GTT GTG AAC CAT GTG ACT TC; *Sqle* forward, ATA AGA AAT GCG GGG ATG TCA C; reverse, ATA TCC GAG AAG GCA GCG AAC; *Acly* forward, AGG AAG TGC CAC CTC CAA CAG T; reverse, CGC TCA TCA CAG ATG CTG GTC A; *Slc25a1* forward, GGA GAG GAC TAT TGT GCG GTC T; reverse, CCC GTG GAA AAA TCC TCG GTA C; *Ldlr* forward, TGA CTC AGA CGA ACA AGG CTG; reverse, ATC TAG GCA ATC TCG GTC TCC; *Idol* forward, TGC AGG CGT CTA GGG ATC AT; reverse, GTT TAA GGC GGT AAG GTG CCA; *Abca1* forward, AAA ACC GCA GAC ATC CTT CAG; reverse, CAT ACC GAA ACT CGT TCA CCC; *Abcg1* forward, CTT TCC TAC TCT GTA CCC GAG G; reverse, CGG GGC ATT CCA TTG ATA AGG; *Actin* forward, CAG CTT CTT TGC AGC TCC TT; reverse, CAC GAT GGA GGG GAA TAC AG; *Ifng* forward, ATG AAC GCT ACA CAC TGC ATC; reverse, CCA TCC TTT TGC CAG TTC CTC; *Il4* forward, AGG AGC CAT ATC CAC GGA TG; reverse, ACA GAC GAG CTC ACT CTC TG. *Pparg* forward,

GTA CTG TCG GTT TCA GAA GTG CC; reverse, ATC TCC GCC AAC AGC TTC TCC T; *Pparg1* forward, AGA AGC GGT GAA CCA CTG ATA TTC; reverse, AGA GGT CCA CAG AGC TGA TTC C; *Pparg2* forward, TGC TGG TGA TCA GAA GGC TG; reverse, AGT GGT TCA CAG CTT CTT TCA A.

qPCR primers for human genes are listed as follows: Srebf1 forward, CAA GGC CAT CGA CTA CAT T; reverse, TTG CTT TTG TGG ACA GCA GT; Actin forward, AGA GCT ACG AGC TGC CTG AC; reverse, AGC ACT GTG TTG GCG TAC AG.

ChIP-qPCR primers are listed as follows: P1 forward, ATC GGA ACG AGT TAA TAC CA; reverse, AGT TAC CTG TGT CAG CAG; P2 forward, AGC AAG GCA ATC CAG AGA; reverse, TTT ACC CTG TGC GGA AAG; P3 forward, TTC CTT GTC TTC CTT CCT T; reverse, ATC TGC CTT GTT CTG GAA; P4 forward, GAC AGG CTA TTT GAG TTT; reverse, AAA TTA CAC AGG AGT TGG; P5 forward, AAT GTG TCC TGG TGA AGC; reverse, GAT ACC TCG GCT GCT TTC; P6 forward, TTA CCT GTT CCT AAG TCA; reverse, AGA AGA GAA GAG AAG AGA; P7 forward, TTG ACT TTG AGA TTC ACA CTT; reverse, CTA CCA TTG GAG CAG AGA; P8 forward, CCA TGC TTC TCT AAC CTC TC; reverse, CTC CAC TAT GTG CCT CTG; P9 forward, CCT TTA ATC TAA CGA TGT CT; reverse, CTT GGC TTC TTC TGT ATC; Nr1h3 promoter region forward, GAG AGG TTC AGA GAC ATT; reverse, TCC GTG TTA CAG TTA CAA; 293T Srebf1 promoter forward, TGT CCT CAA GAG GCC AAT CT; reverse, ACT GCT GGG CCA GTT ACA TC.

Supplementary Figures

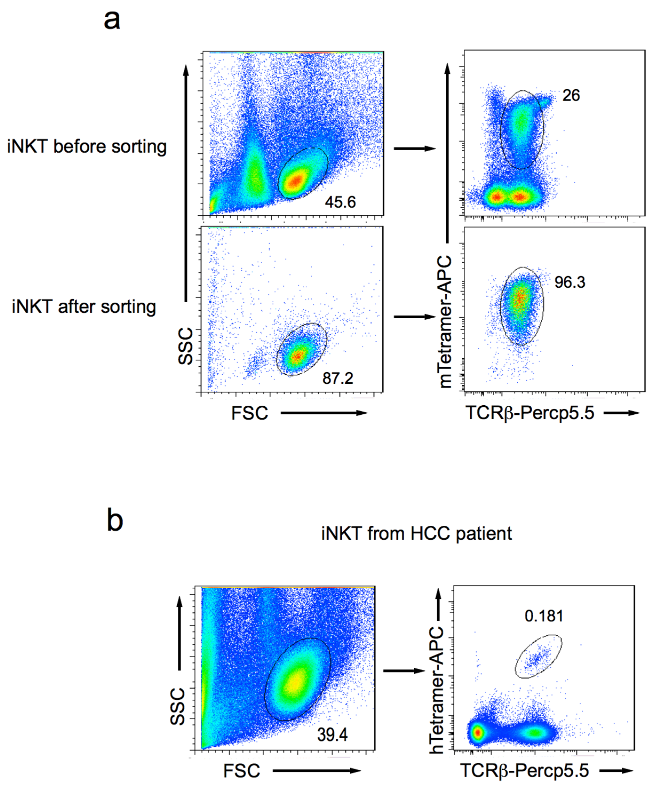

Supplementary Figure 1. Gating and sorting strategy for mouse and human iNKT cells. (a) mCD1d-pbs57 tetramer staining of iNKT cells from livers of *Vα14* Tg.*cxcr6*$^{gfp/+}$ mice before and after sorting. Same gating strategy was used to identify mouse iNKT cells in this study. (b) hCD1d-pbs57 tetramer staining of iNKT cells from HCC patient. Gating strategy for human iNKT cells in Fig. 5. Data are representative of ten independent experiments (a, b). Source data are provided as a Source Data file.

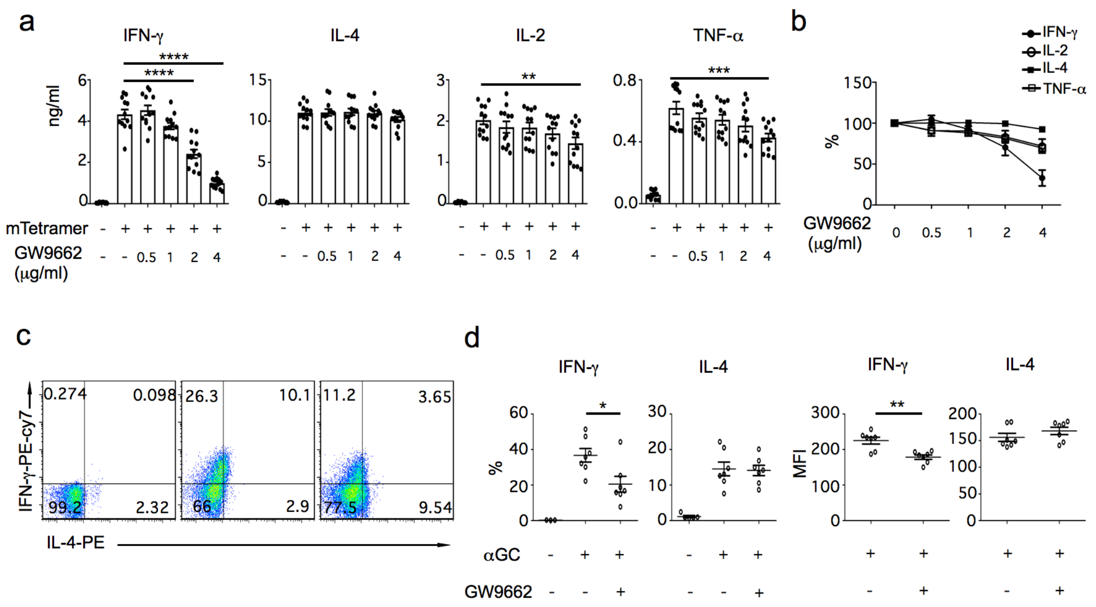

Supplementary Figure 2. Influences of GW9662 on iNKT cell functions *in vitro* and *in vivo*. (a, b) Influences of GW9662 on IFN-γ, IL-2, TNF-α and IL-4 production from iNKT cells activated by plate-coated mCD1d-PBS57 tetramer. (c, d) Influences of GW9662 on intracellular IFN-γ and IL-4 (c), percentages of IFN-γ^{+} iNKT cells, IL-4^{+} iNKT cells, and mean fluorescence intensity of anti-IFN-γ and anti-IL-4 (d) in hepatic iNKT cells activated by αGC *in vivo*. Data are representative of three independent experiments (c), or are means ± SEM of twelve biological replicates (a, b), or seven mice (d), pooled from three to five independent experiments. Data were analyzed by unpaired Student's t-test (a) or Mann-Whitney test (d). *P < 0.05, **P < 0.01, ***P < 0.001, ****P < 0.0001. Source data are provided as a Source Data file.

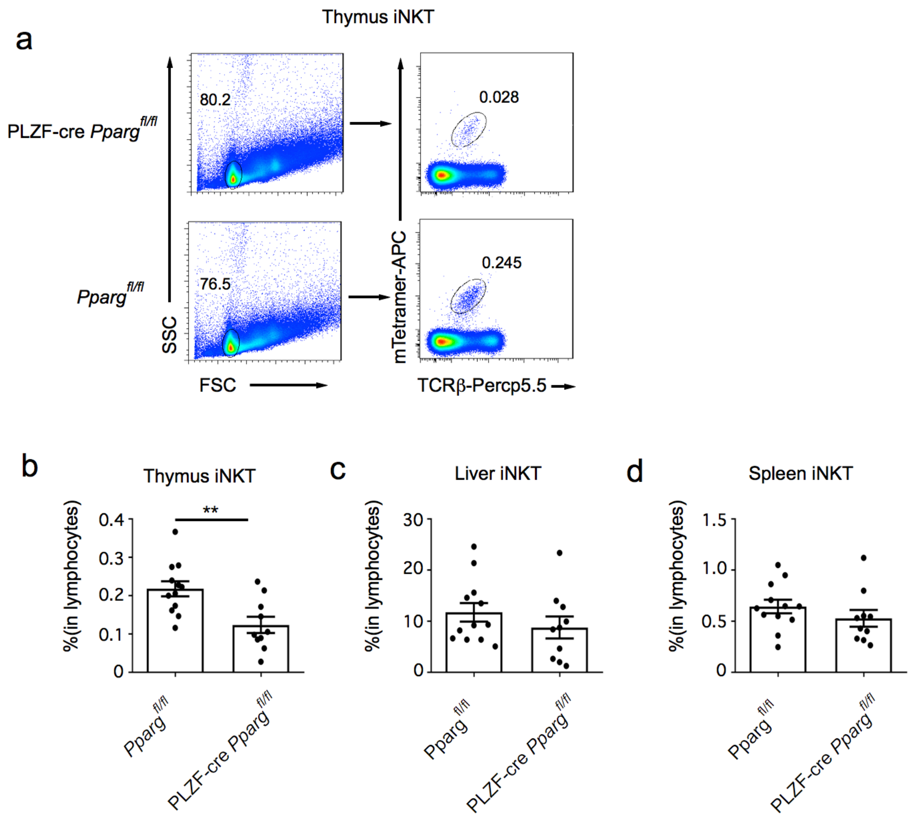

Supplementary Figure 3. Development of iNKT cells in PLZF cre *Pparg*$^{fl/fl}$ mice. (a-d) CD1d-pbs57 tetramer staining of iNKT cells from thymuses (a) and percentages of iNKT cells from thymuses (b), livers (c), and spleens (d) of PLZF-cre *Pparg*$^{fl/fl}$ mice and *Pparg*$^{fl/fl}$ mice. Data are representative of (a) or are means ± SEM of ten to twelve mice per group (b-d). Data were analyzed by Mann-Whitney test (b-d). **P < 0.01. Source data are provided as a Source Data file.

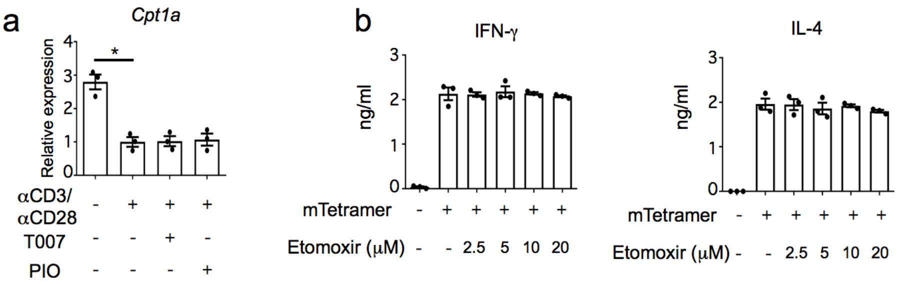

Supplementary Figure 4. Inhibition of β-oxidation shows no influence on iNKT cell functions. (a) mRNA of *Cpt1a* in iNKT cells activated by plate-coated anti-CD3 and anti-CD28 for 24 hours with or without T007, or PIO. (b) IFN-γ and IL-4 production in iNKT cells activated by plate-coated mCD1d-PBS57 tetramer in the absence or presence of Etomoxir. Data are means ± SEM of three independent experiments (a, b). Data were analyzed by Mann-Whitney test (a, b). *P < 0.05. Source data are provided as a Source Data file.

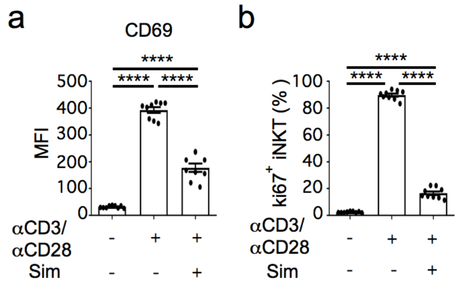

Supplementary Figure 5. Cholesterol promotes activation and proliferation of iNKT cells. (a) Surface CD69 on iNKT cells after activating by plate-coated anti-CD3 and anti-CD28 for 24 hours in the absence or presence of Sim. Unstimulated iNKT cells were used as negative controls. (b) Frequencies of Ki67^{+} iNKT cells after activating with plate-coated anti-CD3 and anti-CD28 for 2 days with or without Sim. Data are means ± SEM of nine biological replicates pooled from three independent experiments (a, b). Data were analyzed by unpaired Student's t-test (a, b). ****P < 0.0001. Source data are provided as a Source Data file.

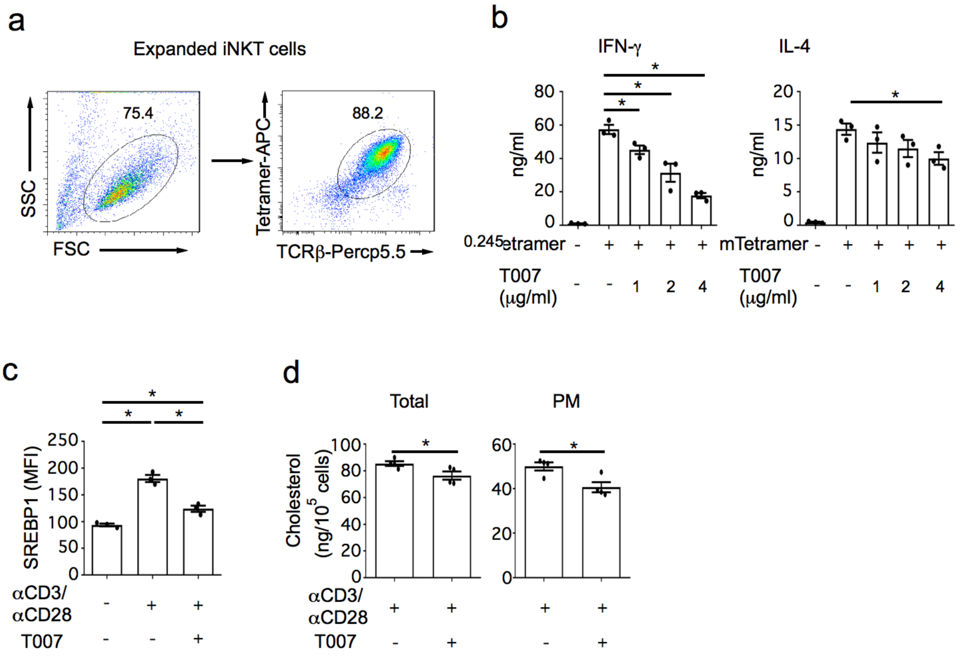

Supplementary Figure 6. Expanded iNKT cells respond to T007 similarly as fresh iNKT cells. (a) Purity of *in vitro* expanded iNKT cells. (b) IFN-γ and IL-4 production from expanded iNKT cells activated by plate-coated mCD1d-PBS57 tetramer in the absence or presence of T007. (c) Level of total SREBP1 protein in expanded iNKT cells activated by plate-coated anti-CD3 and anti-CD28 overnight in the absence or presence of T007. (d) Total cholesterol or plasma membrane cholesterol in expanded iNKT cells activated by anti-CD3 plus anti-CD28 for 24 hours with or without T007. Data are representative of ten independent experiments (a), or are means ± SEM of three to four independent experiments (b-d). Data were analyzed by Mann-Whitney test (b-d). $^*P < 0.05$. Source data are provided as a Source Data file.

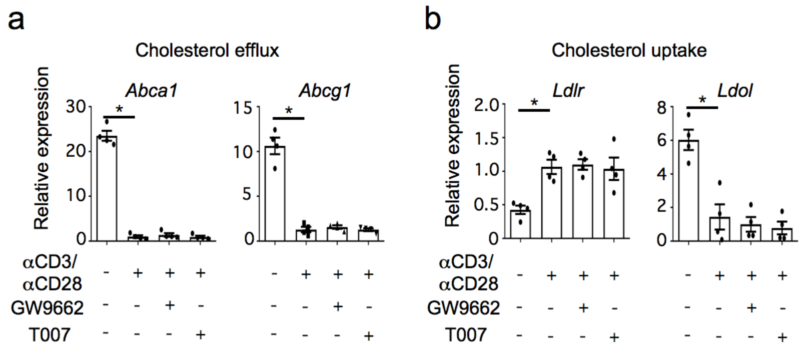

Supplementary Figure 7. PPARγ shows no influence on cholesterol transport. (a, b) Influences of GW9662 and T007 on mRNA of genes regulating cholesterol efflux (a) and uptake (b). Data are means ± SEM of four independent experiments (a, b). Data were analyzed by Mann-Whitney test (a, b). *P < 0.05, **P < 0.01, ***P < 0.001. Source data are provided as a Source Data file.

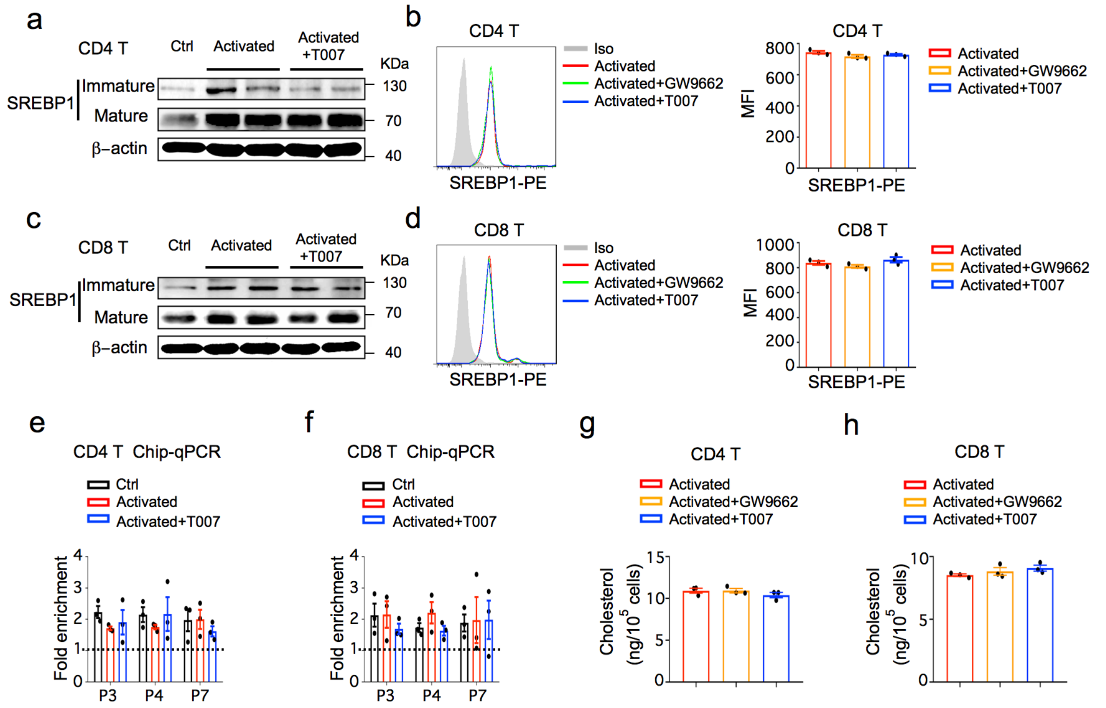

Supplementary Figure 8. PPARγ does not regulate expression of SREBP1 in conventional T cells. (a-d) Mature and immature SREBP1 levels, and total SREBP1 protein levels in CD4^{+} T (a, b) and CD8^{+} T cells (c, d) activated by plate-coated anti-CD3 plus anti-CD28 in the presence or absence of T007. (e, f) Influences of T007 on binding of PPARγ to indicated fragments in promoter region of *Srebf1* in CD4^{+} T cells and CD8^{+}T cells after activating with plate-coated anti-CD3 plus anti-CD28 for 15 hours. (g, h) Cholesterol in CD4^{+} T cells and CD8^{+} T cells activated by anti-CD3 plus anti-CD28 for 24 hours with or without GW9662, T007. Data are representative of three independent experiments (a-d), or are means ± SEM of three independent experiments (b, d-h). Data were analyzed by Mann-Whitney test (b, d-h). Source data are provided as a Source Data file.

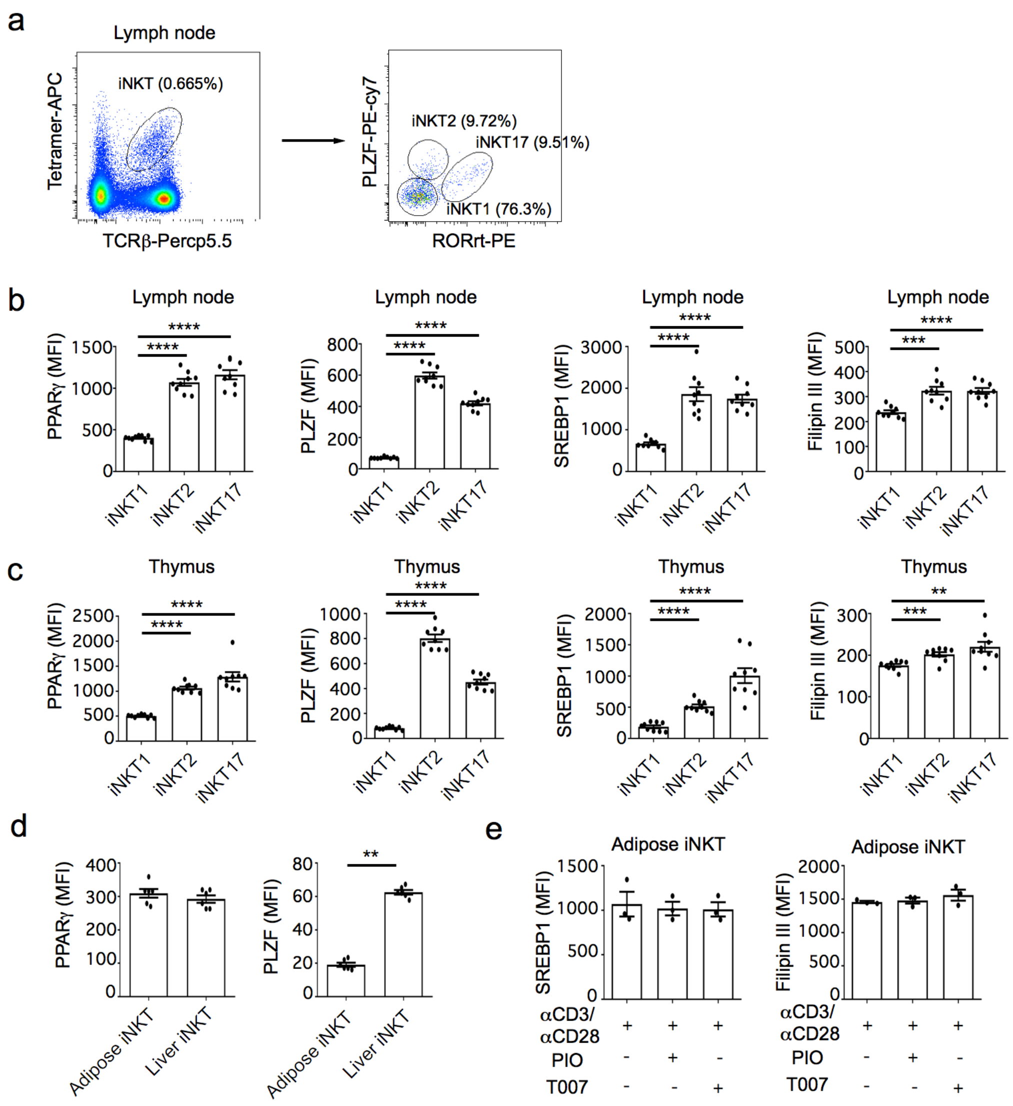

Supplementary Figure 9. PPARγ, PLZF, SREBP1, and cholesterol in distinct iNKT subsets. (a) Gating strategy for iNKT1, iNKT2, and iNKT17 cells. Cells shown were pooled from 3 lymph nodes. (b, c) Protein levels of PPARγ, PLZF, SREBP1, and Filipin III staining in iNKT1, iNKT2, and iNKT17 cells in lymph node (b) and thymus (c). (d, e) PPARγ (d) and PLZF (e) expression in adipose tissue and liver iNKT cells. (f) Influences of T007 and PIO on SREBP1 expression (left) and Filipin III staining (right) in adipose tissue iNKT cells activated by plate-coated anti-CD3 and anti-CD28 for 15 hours. Data are representative of three experiments (a), or are

means ± SEM of nine mice (b-c), six mice (d), pooled from three independent experiments, or are means ± SEM of three independent experiments (e). Data were analyzed by unpaired Student's t-test (b-c) or Mann-Whitney test (d, e). $^{**}P < 0.01$, $^{***}P < 0.001$, $^{****}P < 0.0001$. Source data are provided as a Source Data file.

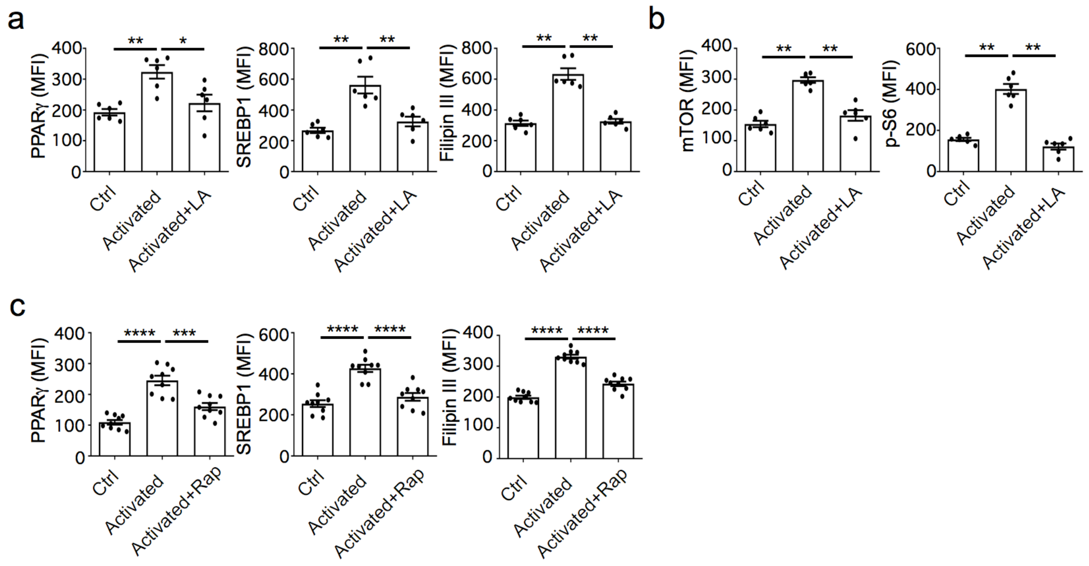

Supplementary Figure 10. Lactic acid inhibits PPARγ expression in iNKT cells through mTORC1 pathway. (a) Protein levels of PPARγ, SREBP1, and Fillipin III staining in iNKT cells activated by plate-coated anti-CD3 plus anti-CD28 for 15 hours in the presence or absence of lactic acid (20 μM). (b) Protein levels of mTOR and p-S6 in iNKT cells activated by plate-coated anti-CD3 plus anti-CD28 for 15 hours in the presence or absence of latic acid (20 μM). (c) Protein levels of PPARγ, SREBP1, and Fillipin III in iNKT cells activated by plate-coated anti-CD3 plus anti-CD28 for 15 hours in the presence or absence of rapamycin (4 nM). Data are means ± SEM of six biological replicates (a, b) or nine biological replicates (c), pooled from three independent experiments. Data were analyzed by Mann-Whitney test (a, b) or unpaired Student's t-test (c). *P < 0.05, **P < 0.01, ***P < 0.001, ****P < 0.0001. Source data are provided as a Source Data file.

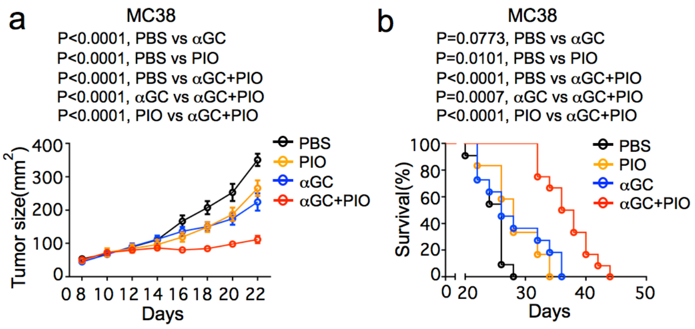

Supplementary Figure 11. (a, b) Tumor size (a) and survival rate (b) of MC38 tumor-bearing mice receiving indicated treatments (PBS, n = 11; PIO, n = 12; αGC, n = 11; αGC+PIO, n = 12). Error bars represent SEM. Data were analyzed by two-way ANOVA (a), or log-rank test (b). Source data are provided as a Source Data file.
