# Supplementary material for: Impaired lipid biosynthesis hinders anti-tumor efficacy of intratumoral iNKT cells
Source: Nat Commun. 2020 Jan 23;11:438. doi: 10.1038/s41467-020-14332-x (PMC6978340; doi:10.1038/s41467-020-14332-x)
Supplement: Supplementary file 3 — Reporting Summary [file 41467_2020_14332_MOESM3_ESM.pdf]

nature research

Corresponding author(s): Li Bai

Last updated by author(s): Dec 24, 2019

Reporting Summary

Nature Research wishes to improve the reproducibility of the work that we publish. This form provides structure for consistency and transparency in reporting. For further information on Nature Research policies, see Authors & Referees and the Editorial Policy Checklist.

Statistics

For all statistical analyses, confirm that the following items are present in the figure legend, table legend, main text, or Methods section.

n/a	Confirmed	
☐	☒	The exact sample size (n) for each experimental group/condition, given as a discrete number and unit of measurement
☐	☒	A statement on whether measurements were taken from distinct samples or whether the same sample was measured repeatedly
☐	☒	The statistical test(s) used AND whether they are one- or two-sided<br>*Only common tests should be described solely by name; describe more complex techniques in the Methods section.*
☒	☐	A description of all covariates tested
☐	☒	A description of any assumptions or corrections, such as tests of normality and adjustment for multiple comparisons
☐	☒	A full description of the statistical parameters including central tendency (e.g. means) or other basic estimates (e.g. regression coefficient) AND variation (e.g. standard deviation) or associated estimates of uncertainty (e.g. confidence intervals)
☐	☒	For null hypothesis testing, the test statistic (e.g. F, t, r) with confidence intervals, effect sizes, degrees of freedom and P value noted<br>*Give P values as exact values whenever suitable.*
☒	☐	For Bayesian analysis, information on the choice of priors and Markov chain Monte Carlo settings
☒	☐	For hierarchical and complex designs, identification of the appropriate level for tests and full reporting of outcomes
☐	☒	Estimates of effect sizes (e.g. Cohen's d, Pearson's r), indicating how they were calculated

Our web collection on statistics for biologists contains articles on many of the points above.

Software and code

Policy information about availability of computer code

Data collection	We have clearly stated all software collecting the data in the methods.
Data analysis	We used the GraphPad Prism to analyze the data.

For manuscripts utilizing custom algorithms or software that are central to the research but not yet described in published literature, software must be made available to editors/reviewers. We strongly encourage code deposition in a community repository (e.g. GitHub). See the Nature Research guidelines for submitting code & software for further information.

Data

Policy information about availability of data

All manuscripts must include a data availability statement. This statement should provide the following information, where applicable:

- Accession codes, unique identifiers, or web links for publicly available datasets
- A list of figures that have associated raw data
- A description of any restrictions on data availability

There is no restriction in the availability of data in the study. The source data are provided as a Source Data file.

Field-specific reporting

Please select the one below that is the best fit for your research. If you are not sure, read the appropriate sections before making your selection.

☒ Life sciences ☐ Behavioural & social sciences ☐ Ecological, evolutionary & environmental sciences

For a reference copy of the document with all sections, see nature.com/documents/nr-reporting-summary-flat.pdf

Life sciences study design

All studies must disclose on these points even when the disclosure is negative.

Sample size	Experiments were repeated at least three times. Data were pooled from independent experiments in most of the figures with sample size at least of 5 for mouse experiments, human samples, and ChIP experiments. In vitro experiments had a large sample size of 9 or more. Same sample size is typically used in the literatures.
Data exclusions	Only animals that got unhealthy were excluded from the study.
Replication	Experiments were repeated at least three times.
Randomization	All the mice used in this study were grouped randomly.
Blinding	The investigators were blinded to group allocation during data collection and analysis.

Reporting for specific materials, systems and methods

We require information from authors about some types of materials, experimental systems and methods used in many studies. Here, indicate whether each material, system or method listed is relevant to your study. If you are not sure if a list item applies to your research, read the appropriate section before selecting a response.

Materials & experimental systems

n/a	Involved in the study	
☐	☒	Antibodies
☐	☒	Eukaryotic cell lines
☒	☐	Palaeontology
☐	☒	Animals and other organisms
☐	☒	Human research participants
☒	☐	Clinical data

Methods

n/a	Involved in the study	
☒	☐	ChIP-seq
☐	☒	Flow cytometry
☒	☐	MRI-based neuroimaging

Antibodies

Antibodies used	Antibodies used in this study are shown in Materials and Methods.
Validation	The information for the antibodies used are shown in Materials and Methods. The companies provide validation data for staining of cells.

Eukaryotic cell lines

Policy information about cell lines

Cell line source(s)	The 293T cell line was provided by cooperator.
Authentication	The 293T cell line is commonly used in many references.
Mycoplasma contamination	We had not tested mycoplasma contamination.
Commonly misidentified lines (See ICLAC register)	We had not used any commonly misidentified cell lines.

Animals and other organisms

Policy information about studies involving animals; ARRIVE guidelines recommended for reporting animal research

Laboratory animals	Mouse strain and age are reported in methods section. Both male and female mice were used.
Wild animals	This study did not involve wild animals.
Field-collected samples	This study did not involve samples collected from the field.
Ethics oversight	All animal procedures were approved by the Animal Care and Use Committee of University of Science and Technology of China, and all experiments were performed in accordance with the approved guidelines.

Note that full information on the approval of the study protocol must also be provided in the manuscript.

Human research participants

Policy information about studies involving human research participants

Population characteristics	Clinical tumor biospecimens were obtained from HCC patients.
Recruitment	The participants were recruited randomly.
Ethics oversight	Clinical tumor biospecimens were obtained from HCC patients in accordance with protocols approved by the Biomedical Ethics Committee of Anhui Medical University. Informed consent was obtained from all subjects.

Note that full information on the approval of the study protocol must also be provided in the manuscript.

Flow Cytometry

Plots

Confirm that:

☒ The axis labels state the marker and fluorochrome used (e.g. CD4-FITC).

☒ The axis scales are clearly visible. Include numbers along axes only for bottom left plot of group (a 'group' is an analysis of identical markers).

☒ All plots are contour plots with outliers or pseudocolor plots.

☒ A numerical value for number of cells or percentage (with statistics) is provided.

Methodology

Sample preparation	Sample preparation is described in Materials and Methods-Cell isolation and activation.
Instrument	Samples were acquired by a BD FACSVerse flow cytometry.
Software	Data were analyzed with FlowJo software (TreeStar).
Cell population abundance	Purity of cells after sorting was checked by FACS, and is shown in Supplementary Figure 1.
Gating strategy	Gating strategy is shown in the Supplementary Figure 1.

☒ Tick this box to confirm that a figure exemplifying the gating strategy is provided in the Supplementary Information.
